# Effect of Chest Proprioceptive Neuromuscular Facilitation on the Respiratory Status of Post-stroke Survivor: A Systematic Review

**DOI:** 10.7759/cureus.71594

**Published:** 2024-10-16

**Authors:** Rachel Dsilva, Suraj Kanase

**Affiliations:** 1 Neuro Physiotherapy, Krishna Vishwa Vidyapeeth (Deemed to be University), Karad, IND

**Keywords:** chest pnf, physiotherapy, pnf, stroke, systematic review

## Abstract

Respiratory complications are one of those complications that can determine a patient’s prognosis in the acute state and are a commonly neglected complication in the chronic state. Respiratory complications can range from improper ventilation profusion to thick copious secretion which can slow the prognosis. Respiratory complications need to be addressed in order to ensure a good ventilation perfusion and in the long run increase the patient’s quality of life. Chest physiotherapy helps in centralizing the secretions and aids in the removal of the secretions. There are many conventional techniques to aid the betterment of the respiratory position such as chest percussion, vibration, and shaking. The purpose of this study was to find the effectiveness of chest proprioceptive neuromuscular facilitation (PNF) in improving the pulmonary complications of post-stroke patients. The following articles have reviewed a more advanced technique to help remove secretion and increase vital capacity. All the reviewed articles used the chest PNF techniques and their variations as they provide tactile stimulus to lead to muscle contraction.

## Introduction and background

Stroke is one of the leading causes of worldwide disability. It occurs as a result of a vascular cause leading to an injury to the central nervous system [1]. There is an increasing burden of stroke in India, Stroke being the fifth foremost cause of disability and the fourth chief cause of death. The incidence of stroke in India ranges between 105 and 152/100,000 people per year, according to previous research [2]. Overall, 59% of patients die during the first five years and 61% of patients die during the first seven years of stroke which is the exact estimation of mortality in India. The majority of patients who get affected by stroke have a long-term disability and rarely return to full function.

A sudden loss of neurological function caused by an interruption of blood flow to the brain is termed a stroke. A stroke is caused due to a Cerebrovascular accident. It is the event that occurs within the arteries which either can be ischemic that is clot formation, hemorrhagic, thrombotic, or embolic. When disruption of blood flow occurs to the brain tissue, the brain areas get deficient in oxygen and nutrients which are essential, else can cause brain cell death.

Various pathologies can cause different types of strokes. Ischemic stroke occurs when the blood flow to an area gets compromised due to a clot that directly compromises the oxygen and nutrient supply to a particular area. Blockage of the artery is caused by the thickening of the innermost layer of the vessels causing atherosclerosis. Embolic stroke can occur due to a fat or air embolism. When a blood clot or a thrombus gets dislodged from its site of origin it can lead to a thrombotic stroke. Elevated intracranial is a classical sign of hemorrhagic stroke which occurs due to the rupture of a blood vessel causing leaking of blood in and around the brain tissues. A transient ischemic attack is a brief disruption of blood flow to the brain. The neurological symptoms may last for less than 24 hours [3].

One of the major contributing factors of cerebrovascular disease is atherosclerosis. Progressive narrowing of the blood vessels occurs due to the deposition of plaque with an accumulation of lipids, fibrin, and complex carbohydrates. According to the American Heart Association, the warning signs of stroke are sudden numbness or weakness of one side of the body, sudden confusion, trouble speaking or understanding, sudden trouble seeing in one or both eyes, sudden loss of balance or coordination, and severe headache. Ischemic cascade sets in motion when there is an interruption of blood flow to a particular area. The ischemic core area gets affected the most as the neurons in this area quickly start to die, while the surrounding penumbra can survive a little longer. A progressive disturbance of energy mobilization and anoxic depolarization is caused by the release of excessive neurotransmitters such as glutamate and aspartate [4].

Post-stroke, one of the major health issues faced by the patients is hemiplegia or hemiparesis. Many other clinical features can be seen in the patient such as altered consciousness, cognitive dysfunction, speech disorders, dysphagia, and cardiovascular and pulmonary dysfunction [3].

Based on the type of stroke that the patient has undergone, the management will vary such as administering tPA to the patient or surgical intervention. Following this the patient will be admitted to intensive care units. For a good prognosis, various interdisciplinary coordination and treatment are required [4]. Early Physiotherapy intervention plays an important role in a better prognosis.

A hospital stay can be a harbor of complications for the patient. With the respiratory system of the patient already compromised that is respiratory muscle weakness, and inability to expel secretions, it is easy for the patient to acquire respiratory complications. Decreased perfusion rate, decreased lung volume altered chest wall excursion are common conclusions [3].

Reduction of cerebral edema, absorption of damaged brain cells, improved cellular metabolism, and local blood circulation allow intact neurons to perform their function that were previously inhibited [4]. This sets a pace for the recovery and to ensure a good prognosis one has to prevent further complications. With conservative neurophysiotherapy rehabilitation, one has to pay keen attention to the rehabilitation of pulmonary functions. Chest percussion, vibrations, and shaking techniques need to be given to mobilize and centralize the secretions. Once the secretions are bought medially depending on the patient's consciousness status the secretions need to be expelled. Along with the above-stated techniques, proprioceptive neuromuscular facilitation (PNF) techniques can be administered to the patient to aid their recovery.

When an external proprioceptive and tactile stimulus is used it produces a reflexive movement response to assist respiration. This is the basic principle behind neurophysiological facilitation, which causes involuntary cough response directly reducing the amount of suctioning required. These techniques can also alter the level of consciousness in some unconscious patients. This treatment has also been shown beneficial to increase vital capacity, peak expiratory flow, and expiratory reserve volume [5]. There is also a note of increased transcutaneous oxygen saturation due to the applications of PNF which can be a great benefit for ICU patients [6]. Various techniques can be given to the patient such as intercostal stretch, anterior stretch basal lift, vertebral pressure, perioral pressure, etc. [7]. 

In intercostal stretch, there is a gradual increase in inspiratory movement in and around the area where the stretch is maintained. When performed on the lower ribs an increase in epigastric excursion is observed [8]. Due to reflective activation of the diaphragm by the intercostal afferents. In vertebral pressure, the afferent input activates the dorsal intercostal muscles. Also, an increase in epigastric excursion and inspiratory movements in the apical thorax is observed. In the anterior stretch, basal lift as a sustained stretch is maintained, causing the dorsal and lateral muscles to be stretched, increasing the epigastric excursion. Maintained manual pressure causes stimulation of tactile cutaneous receptors which elicits an inspiratory response in the stimulated area. Perioral pressure has a direct link with the sucking swallowing movement and indirectly causes increased epigastric excursion. Co-contraction of the abdomen activates the diaphragm as there is an increased tone of the abdomen muscles. This technique can also initiate a cough reflex when there are retained secretions [9].

In numerous previous literature, there have been several interventions for reducing hospital stays and ensuring a good prognosis along the course of the whole treatment [10]. Many studies revealed the interventions to treat the respiratory component that is affected by stroke. They reported the effectiveness of chest PNF to improve inspiratory volume, epigastric excursion and expel secretions [11]. Therefore, the purpose of this study was to systematically review the scientific literature in order to deliver more transparency on the preferred chest PNF techniques to report pulmonary complications in post-stroke survivors.

## Review

Methods

A systematic review of chest PNF in post-stroke patients was performed. This review was done through an electronic search of relevant articles using Google Scholar, PUBMED, Physiotherapy Evidence Database (PEDro), and Research Gate database from 2013 to December 2022 where in chest PNF in post-stroke patients, pulmonary rehabilitation, and intercostal stretch and chest clearance were used as the MeSH search terms. Articles were selected based on capability, self-awareness, and philosophical practice. In addition to this, appropriate books were also searched.

Search Strategy

Articles were searched from Google Scholar, PubMed, Research Gate, PEDro, Cumulative Index to Nursing and Allied Health Literature (CINAHL) between November 9, 2023 and December 3, 2023. Reference lists for eligible articles were formed and authors were contacted for additional data, unpublished articles, and full-text articles.

Inclusion Criteria

All relevant articles were published from January 2014 to January 2024 addressing the following criteria articles that included chest PNF as an intervention, effect on inspiratory volume in post-stroke patients, effect on epigastric excursion in post-stroke patients and study with acute to sub-acute stage post-stroke.

Exclusion Criteria

**Figure 1:**
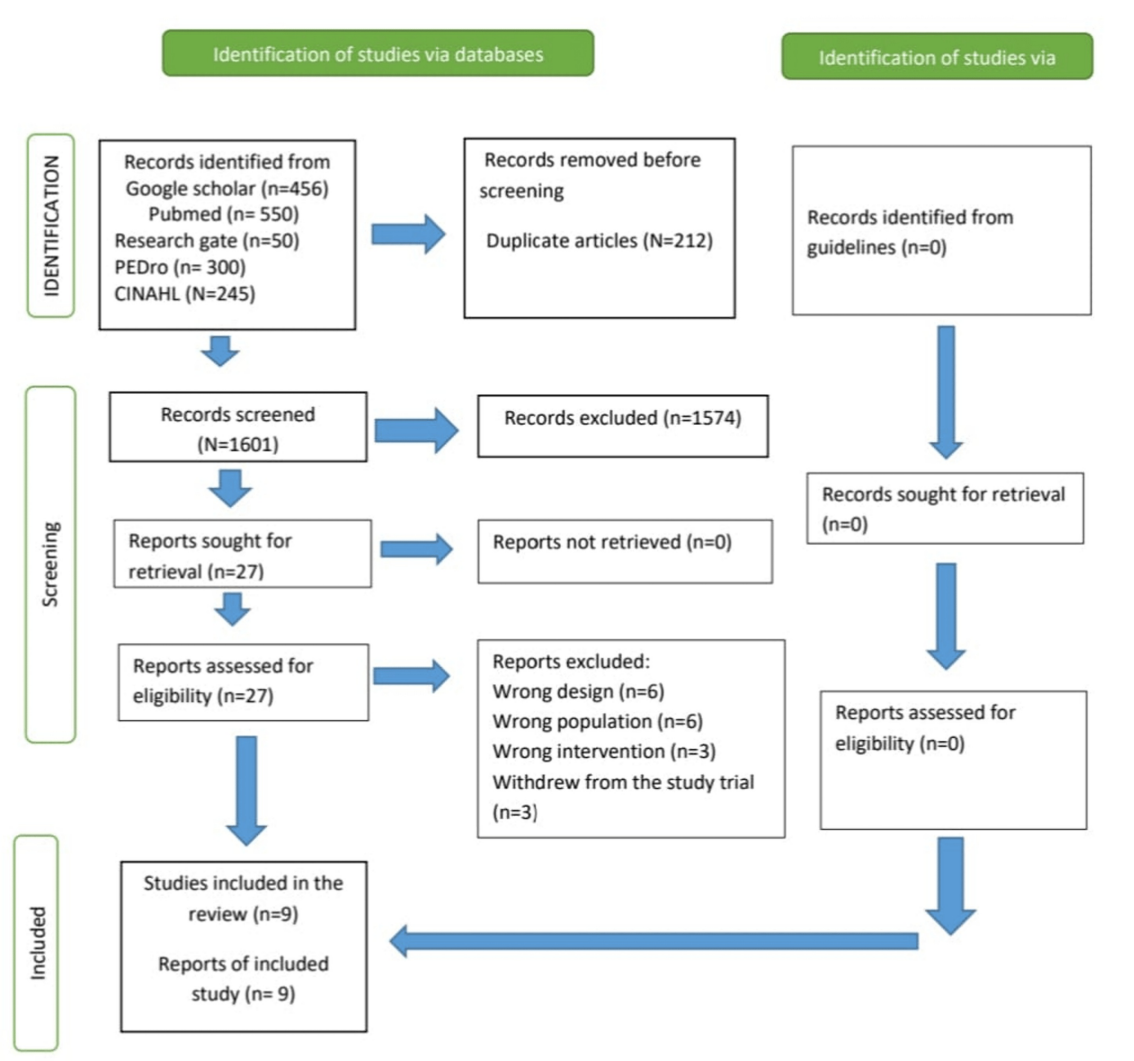
PRISMA flow diagram of the literature search results.

All studies that were duplicates and studies that did not include PNF in the outcome measure, whose population was chronic stroke and inspiratory and epigastric excursion were not used in the outcome measure were excluded. The detailed information on inclusion and exclusion criteria and the search strategy for the articles are illustrated in Figure 1.

Data collection and analysis

Data Extraction

One investigator selected the studies according to the inclusion criteria. The reviewer reviewed the titles and abstract of all the studies. Full texts of appropriate articles were reviewed and were selected if they were in line with the inclusion criteria. The following data was extracted from the related: study design, study population, physiotherapy intervention, physical impact, selected outcome, and key finding.

Assessment of Study’s Risk of Bias

The methodological quantity of the chosen studies was reviewed by two investigators independently and studies which included a high-risk bias was not included.

Results

After searching several databases 1,601 abstracts were extracted. The searches identified 11 appropriately significant studies which met the set inclusion criteria for further analysis.

Demographics

All studies included were of the same study design i.e. randomized control trial. Over 30 samples sizes population was included. These studies showed favorable effects of physiotherapy interventions in post-stroke patients. Studies revealed that chest PNF in post stroke patients improves inspiratory volume, epigastric excursion and aids in removal of secretions to prevent any secondary respiratory complications post stroke. Table 1 contains a summary of included studies.

**Table 1:** Summary of review of literature

Sr. No	Title	Aim of the study	Study design	Sample size	Study duration	Outcome measures	Intervention
1	Immediate effect of intercostal stretch versus anterior basal lift on respiratory rate, chest expansion and spirometry in acute stroke patients (2022) Jadhav et al. [12]	To assess the immediate effect of intercostal stretch versus anterior basal lift on respiratory rate, chest expansion, and intensive spirometry in acute stroke	Randomized controlled trial	44 samples	1 day	Respiratory rate, chest expansion, incentive spirometry	The subjects were divided into 2 groups. Incentive spirometry, respiratory rate, and chest expansion will be taken pre-intervention. Group A was given conventional physiotherapy and intercostal stretch and group B was given conventional physiotherapy and anterior basal lift. 10 repetitions of conventional exercise were given with 1-2 min of rest period. Respiratory PNF was given for 3 breaths with 1 min of rest for 3 repetitions. The total treatment protocol was 45 to 50 mins.
2	Effectiveness of respiratory proprioceptive neuromuscular facilitation (PNF) exercises on respiratory functions in subacute stroke patients of South Gujarat (2021) Valodwala et al. [13]	To check the effectiveness of respiratory proprioceptive neuromuscular facilitation (PNF) exercises on respiratory functions in subacute stroke patients	Randomized controlled trial	34 samples	4 weeks	PFT (FVC, FEV1, FEV1/FVC ratio) were measured using Helios 401	They were allocated into two groups using quasi randomization in which the first patient was allocated to Group A and the second to Group B. Group A was given conventional exercises which included Mat activities (stretching and strengthening), Weight bearing or shifting, and standing lower-extremity exercise in parallel bars and balance activities, Standing, Reaching, Transfers. Group B was given all conventional exercises mentioned in Group A and an additional 20mins of respiratory PNF techniques which include (Peri–oral pressure, Vertebral Pressure, Co –contraction of the abdomen, Intercostal Stretch, Anterior stretch basal lift).
3	Efficacy of chest expansion resistance exercise on respiratory function, trunk control and dynamic balance in patients with chronic stroke (2021) Nair et al. [14]	To see the efficacy of chest expansion resistance exercise on respiratory function, trunk control, and dynamic balance in patients with chronic stroke	Randomized controlled trial	34 samples	4 weeks	Chest expansion, respiratory muscle strength by noninvasive micro-respiratory pressure meter, trunk impairment scale, balance by mini-BESTest	The experimental group received chest expansion resistance exercise (CERE) chest PNF with conventional therapy and the control group received conventional therapy alone. Both groups received therapy four times per week for four weeks.
4	Addition of proprioceptive neuromuscular facilitation to cardiorespiratory training in patients poststroke (2020) de Souza et al. [15]	To assess the effects of respiratory and trunk patterns of CRT associated with PNF on the quality of life, gait, oxygen consumption, respiratory muscle strength, and trunk volumes	Randomized controlled trial	40 patients	1 month	Quality of life, gait, balance, peak oxygen uptake, rib cage compartment volumes, respiratory function, maximal inspiratory and expiratory pressures	The treatment protocol consists of respiratory exercise chest PNF, 30 min of CRT (cycle ergometer) and then repetition of the respiratory exercises, performed three times a week over a period of 20 days.
5	Proprioceptive neuromuscular facilitation for accessory respiratory muscles training in patients after ischemic stroke (2019) Slupska et al. [16]	To study how the pulmonary function Is affected by the PNF of accessory muscles in chronic post-stroke	Randomized controlled trial	30 patients	6 weeks	Electromyography, Barthel scale	The remaining 60 patients were randomly assigned to PNF treatment of respiratory muscles and to just positioning treatment taken as a reference group: 30 patients each.
6	Immediate effects of the respiratory stimulation on ventilation parameters in ischemic stroke survivors (2019) Ptaszkowska et al. [17]	To assess the effect of respiratory stimulation on ventilation parameters in ischemic stroke survivors	Randomized controlled trial	60 patients	1 day	Spirometry test, FVC, FEV1, FEV1/FVC%, PEF	The patients qualified to participate in the study were randomly assigned to 1 of 2 groups in which one-time intervention was performed Group 1 PNF-treated group – in which respiratory stimulation through PNF was used, Group 2 PNF untreated group – in which positioning was used.
7	Effects of chest resistance exercise and chest expansion exercise on stroke patients’ respiratory function and trunk control ability (2015) Song et al. [18]	To examine the effects of chest resistance exercise and chest expansion exercise on stroke patients’ respiratory function and trunk control ability	Randomized controlled trial	40 samples	8 weeks	Forced volume vital capacity (FVC), FEV1, and trunk control ability were measured using the trunk impairment scale (TIS).	Forty patients with stroke were randomly allocated into a chest resistance exercise group (CREG, n = 20) and a chest expansion exercise group (CEEG, n = 20). [Methods] CREG patients underwent chest resistance exercises, and diaphragmatic resistance exercises by way of the proprioceptive neuromuscular facilitation. CEEG patients underwent respiratory exercises with chest expansion in various positions. Both groups received 30 minutes of training per day, five times per week, for eight weeks.
8	The effect of chest expansion resistance exercise in chronic stroke patients: a randomized controlled trial (2015) Kim et al. [19]	To examine the initial effects of chest expansion resistance exercise in chronic stroke patients: a randomized controlled trial	Randomized controlled trial	40 samples	4 weeks	Chest expansion, VC, FVC, FEV1	Forty chronic stroke patients without any respiration-related rehabilitation program experience were randomly and equally allocated to a CERE group (experimental group) and a control group. An ordinary stroke rehabilitation program was performed on the subjects. While the experimental group received a CERE intervention, the control group performed a passive range of motion exercise with automatic instruments.
9	Effect of intercostal stretch technique and anterior basal lift technique on respiratory rate, saturation of peripheral oxygen, and heart rate among ICU patients (2014) Gupta et al. [20]	To assess the effect of the intercostal stretch technique and anterior basal lift technique on respiratory rate, saturation of peripheral oxygen, and heart rate among ICU patients	Randomized controlled trial	30 samples	3 days	Heart rate, oxygen saturation, respiratory rate	Patients are divided into Group A (IC stretch) and Group B (ABL). Patients were given the intervention according to their allocated group for 3 days.

Clinical Characteristics and Management Strategies

Jadhav et al. [12] concluded that anterior stretch basal lift showed significant results than intercostal stretch on improving respiratory rate and chest expansion. Forty-four participants met the inclusion criteria and were divided into two groups. Group A was given conventional therapy with, and addition of intercostal stretch and Group B was given conventional therapy with anterior stretch basal lift. Intensive spirometry, respiratory rate and chest expansion was taken as the outcome measures.

Valodwala et al. [13] conducted in a randomized controlled trial which selected 34 subjected according to the inclusion criteria. They were divided into two groups Group A was given conventional exercises such as mat exercises stretching and strengthening. Group B was given all the exercises of group A and an additional of PNF techniques for 20 mins. FVC, FEV1, FEV1/FVC ratio by PFT were taken as the outcome measures. These were taken on the first day of the treatment and on the last day of treatment. Results revealed that Group B which were given respiratory PNF showed an improvement in respiratory functions.

Nair et al. [14] conducted a comparative study in which a total of 34 participants were selected and were allocated into two groups. The control group received conventional neurological rehabilitation while the experimental group received chest expansion resistance exercise with conventional treatment. Outcome measures used were chest expansion, trunk impairment scale, respiratory muscle strength and mini-BESTest scale. Pre and post assessment was taken on the first and last day of the treatment. Treatment course being four weeks, 16 sessions. Based on the results calculated the experimental group that was given chest expansion resistance exercises showed significant improvement in respiratory function, balance and trunk control in patients with chronic stroke.

de Souza et al. [15] conducted a randomized control trial on 40 patients who were then assembled into four groups to check the effect of trunk and respiratory patterns associated with PNF techniques using oxygen consumption, respiratory muscle strength, quality of life and thoracic volumes as the outcome measures. The four groups were 1) CRT-LL and respiration, 2) CRT-lower limb plus PNF, 3) CRT-upper limb plus PNF, and 4) CRT-upper limb and respiration. The treatment protocol consisted of respiratory training, and 30 mins of CRT cycle ergometer which were performed over a span of 20 days three times per week. Pre and post assessment of the outcome measure were noted. The result concluded after performing this study was that the association of PNF with cardiorespiratory training was an effective and accessible alternative to increase cardiorespiratory functions in post stroke patients.

Slupska et al. [16] conducted a trial including 60 patients who were randomly allocated in two groups of 30. Group A was given PNF treatment of respiratory muscles and Group B was given positioning as a reference group. Pre and post assessment was done with Barthel index and EMG recording of respiratory muscles as the outcome measures. This was to conclude that PNF treatment helped normalize the breathing pattern in stoke patients and reduce the chance of hypoxia.

Ptaszkowska et al. [17] conducted a randomized interventional study which evaluated the respiratory parameters using spirometry, FVC, FEV1, FEV1/ FVC%, PEF as the outcome measures. 60 patients were divided into two groups. Group A underwent PNF treatment for respiratory stimulation. And Group B was treated without PNF and was given positioning. The study concluded that a single treatment of respiratory PNF causes an increase in the air flow and increases the FEV1/FVC%

Song et al. [18] conducted a randomized control trial in which 40 patients met the inclusion criteria. They were further divided into two groups, Group A was given chest resistance exercise, diaphragm resistance exercise by means of PNF. Group B was given chest expansion exercises which included exercises such as respiratory exercises and chest expansion in various positions. Outcome measures were forced volume vital capacity (FVC), FEV1 and trunk impairment scale (TIS). The treatment lasted for eight weeks, 30 min per session five times a week. Results revealed that both groups showed improvement in increasing respiratory function and trunk control, but Group A showed significantly more improvement.

Kim et al. [19] conducted a randomized control trial in which 40 patients with chronic stroke were enrolled in a study that met the inclusion criteria were randomly allocated in two groups. Group A was the experimental group in which chest expansion resistance exercises were given for 20 mins and Group B the control group was given passive range of motion exercises. Chest expansion, Vital capacity, forced vital capacity and forced expiratory volume in one second 10MWT and 6MWT were taken as the outcome measures. It was concluded that chest expansion resistance exercises proved to have a better outcome on the respiratory functions in chronic stroke patients.

Gupta et al. [20] directed a randomized control trial over 30 ICU patients who fulfilled the eligibility criteria. The following patients were randomly assigned to two groups. Group A was given intercostal stretch and Group B was given anterior basal lift twice a day for three days. Outcome measures used were heart rate, oxygen saturation and respiratory rate. Pre and post assessment was done on the first and third day, respectively. It was revealed that intercostal stretch was more effective in reducing heart rate and respiratory rate and improving oxygen saturation.

Discussion

The above analysis is of the past 10 years’ worth of published randomized control trials over the subject of physiotherapy management for respiratory rehabilitation in chronic stroke patients. Randomized controlled trials provided the preponderance of evidence for this study. Nonetheless there were some discrepancies among the study criteria and results. These discrepancies are partially explained by the methodology used to review the selected articles, the type of study chosen and the emphasis of the objectives of the studies. Resulting in a high caliber study the importance of chest PNF techniques in improving the respiratory status of post stroke patients. Many databases were explored to avoid excluding many references and widening the search option and achieving a more specific link between the chosen articles. However, Google Scholar and PubMed were the main databases chosen for majority of the articles while other databases were also explored.

Jadhav et al. [12] concluded in their study that conventional physiotherapy along with respiratory PNF has a positive effect on chest expansion, respiratory rate, and spirometry due to the applied tactile and proprioceptive stimuli that help increase the intrathoracic lung volume and lead to a higher percentage of flow rate. Valodwala et al. [13] came to a conclusion that chest expansion resistance exercises were more effective in improving the trunk function, balance, and mainly respiratory functions in chronic stroke patients as it is based on the principle of PNF in which a stretch reflex resists the change in muscle length thereby facilitating a contraction in the stretched muscle via its muscle spindle. Nair et al. [14] came to the conclusion that respiratory parameters of PFT showed a positive effect due to respiratory PNF as the proprioceptors and mechanoreceptors are activated which showed the effect on muscle spindle and tendon that are sensitive to the forces of respiration which in turn cause an increased coordination in the respiratory muscles. de Souza et al. [15] conducted a study that revealed cardiorespiratory functions can be improved with a combination of cardiorespiratory training and chest PNF as it causes alternating isotonic contractions to occur by the principle of stabilization reversal. Slupska et al. [16] came to a conclusion that PNF treatment can reduce the overactivity of accessory respiratory muscles and can normalize breathing patterns as PNF to the primary muscles of respiration can reduce the bioelectric activity of the accessory muscles and improves lung oxygenation and ventilation. Ptaszkowska et al. [17] concluded that the respiratory parameters can be increased by a single intervention of respiratory stimulation through PNF as it increases muscle expansion in the paretic side which in turn increases the respiratory parameters. Song et al. [18] concluded that chest resistance exercise is more effective in improving the respiratory functions and trunk control as this technique enhances the respiratory muscle strength and endurance causing it to have a direct effect on the respiratory status of the patient. Kim et al. [19] came to a conclusion that chest expansion resistance exercises play an important role in alleviating the respiratory functions in stroke rehabilitation. These techniques facilitate the respiration which increases the air flow that is translated dues to increased chest expansion. Gupta et al. [20] revealed in their study that using intercostal stretch techniques are more effective in reducing respiratory and heart rate and improving the oxygen saturation than the anterior basal lift as intercostal stretch in the alpha motor neuron activity which directly has an effect on the muscle fiber to initiate contraction. However, the study has limitations regarding the specificity of chest PNF techniques. This study focuses on overall techniques and is not pertaining to single specific techniques and its impact on the outcome.

Summary

A total of nine articles were included. The study design for all the reviewed articles were consistent, i.e., randomized controlled trial. Three articles that were included had a study duration for one day; the remaining studies had a duration of minimum of three weeks. One article used EMG as their outcome measure. All studies had a component of chest PNF in the intervention.

## Conclusions

The above articles mentioned were examined thoroughly and gave a summary of the effect of chest PNF on the respiratory status of a post-stroke survivor. The studies that were evaluated showed significant results in increasing the tidal volume and oxygen saturation (SPO_2_) levels while maintaining the respiratory rate in post-stroke patients due to chest PNF. Air exchange was increased by various techniques such as intercostal stretch, anterior basal lift, perioral pressure, and intra-abdominal pressure maybe be useful in improving the respiratory status of post-stroke patients. These techniques can be used to facilitate the diaphragm muscle and inhibit the use of accessory ones for respiration which will facilitate the correct pattern of breathing. Inter-abdominal pressure can be used to facilitate a cough reflex and aid in the expulsion of secretions.
